# The role of TREX in gene expression and disease

**DOI:** 10.1042/BCJ20160010

**Published:** 2016-09-27

**Authors:** Catherine G. Heath, Nicolas Viphakone, Stuart A. Wilson

**Affiliations:** Department of Molecular Biology and Biotechnology, The University of Sheffield, Firth Court, Western Bank, Sheffield S10 2TN, U.K.

**Keywords:** mRNA export, nucleocytoplasmic, NXF1, RNA processing, THO, transport

## Abstract

TRanscription and EXport (TREX) is a conserved multisubunit complex essential for embryogenesis, organogenesis and cellular differentiation throughout life. By linking transcription, mRNA processing and export together, it exerts a physiologically vital role in the gene expression pathway. In addition, this complex prevents DNA damage and regulates the cell cycle by ensuring optimal gene expression. As the extent of TREX activity in viral infections, amyotrophic lateral sclerosis and cancer emerges, the need for a greater understanding of TREX function becomes evident. A complete elucidation of the composition, function and interactions of the complex will provide the framework for understanding the molecular basis for a variety of diseases. This review details the known composition of TREX, how it is regulated and its cellular functions with an emphasis on mammalian systems.

## Introduction

**Figure 1.**
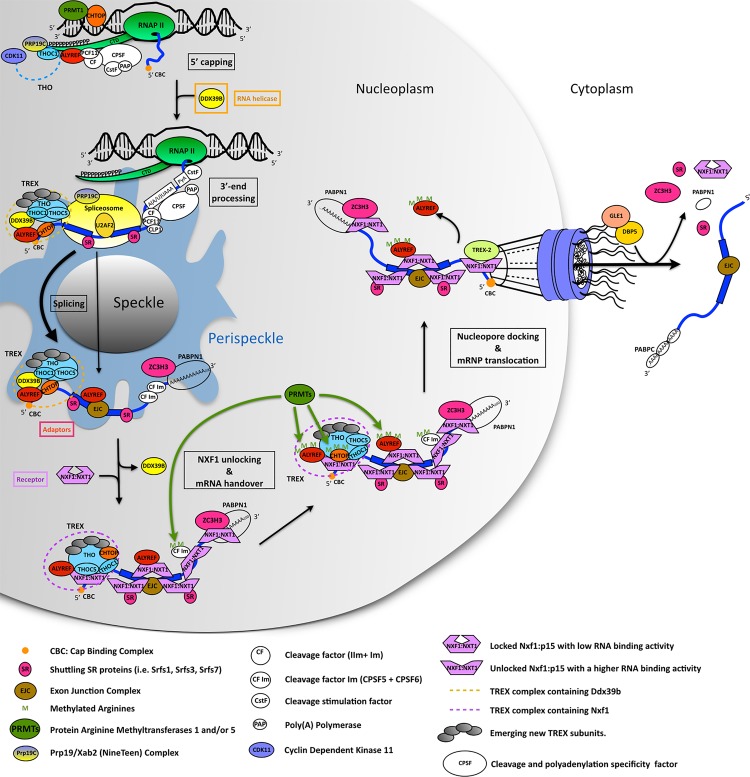
Overview of NXF1- and TREX-dependent mRNP formation and nucleocytoplasmic transport. During gene expression, the TREX complex is recruited co-transcriptionally by a direct interaction with the phosphorylated C-terminal domain (CTD) of the large subunit of RNA polymerase II and via the PRP19:U2AF2 complex. Each processing step undergone by the pre-mRNA (5′-capping, co- and posttranscriptional splicing, 3′-end processing) acts as a trigger for TREX assembly and deposition along the transcript. Once bound to the mRNA, the combined action of adaptor (red and pink) and co-adaptor proteins (e.g. THOC5, CHTOP and CPSF6) recruit the NXF1:NXT1 heterodimer and cause a conformation change in NXF1, allowing exposure of its RBD for interaction with mRNA. In turn, the adaptor protein hands the mRNA over to NXF1. This process is promoted by protein arginine methyltransferase 1 (PRMT1) through arginine methylation of both the co-adaptor CHTOP, facilitating its interaction with NXF1, and the adaptor ALYREF, which reduces its RNA-binding activity enabling RNA handover to NXF1. Additional adaptor proteins, such as SR proteins and ZC3H3, can also recruit NXF1 to mRNA. Subsequently, direct interactions of NXF1 with the TREX-2 complex and nucleoporins allow the mRNP to dock at the nuclear pore, thus promoting export of the mRNP. At the nuclear pore export, adaptors are released from the mRNP, this is predicted to revert NXF1 to a low-affinity RNA-binding state that primes it for release from the mRNP. DBP5 and GLE1 trigger the release of NXF1 from the mRNP on the cytoplasmic side of the nuclear pore for recycling back to the nucleus.

Protein-coding genes transcribed by RNA polymerase II (Pol II) give rise to RNA molecules termed pre-mRNAs. During and/or after their synthesis, these precursors undergo a series of three main processing steps. Their 5′ end receives an m^7^G cap structure, shortly after the initiation of transcription, which protects the nascent pre-mRNA from degradation and plays a role in further stages of mRNA maturation [1]. The introns are excised during splicing which generally occurs co-transcriptionally, although approximately 20% of splicing events take place following transcription [2]. The 3′-end of the pre-mRNA is processed by endonucleolytic cleavage and polyadenylation, leaving the transcript with a polyadenosine (poly(A)) tail [3]. Following these processing events, the mRNA is exported from the nucleus through the nuclear pore complex to the cytoplasm where it can be translated into proteins (Figure 1).

From its site of synthesis until it reaches the nuclear pore, the future mRNA associates with numerous proteins to form a messenger ribonucleoprotein (mRNP). Throughout its nuclear odyssey, the mRNP composition is modified by the various remodeling events that accompany each maturation stage (Figure 1). The completion of each processing step results not only in a modified RNA molecule but also in the association of a specific set of proteins with the RNA. These two hallmarks of a mature mRNA are monitored by quality control mechanisms that trigger the degradation of defective mRNAs either in the nucleus by the nuclear exosome [4] or in the cytoplasm via the NMD pathway [5]. The expression of a functional protein thus relies on a properly synthesized and processed mRNA transcript packaged with the appropriate group of proteins.

A key player in mRNP biogenesis and maturation is the TRanscription and EXport (TREX) complex. TREX is conserved across a wide range of organisms including *Saccharomyces cerevisiae*, *Drosophila*, *Arabidopsis*, *Xenopus* and humans [6–10], indicating its key physiological importance. Components of TREX are functionally linked to each mRNA processing step, and this is reflected by their physical association with protein complexes such as the 5′ cap-binding complex (CBC), the exon junction complex (EJC) and 3′-end processing factors [11–13] (Figure 1). Therefore, TREX probably acts as an interface for these various processes to help maintain high fidelity of the gene expression process. In this review, focused primarily on mammalian TREX, we present the known composition with an emphasis on its dynamic organization uncovered over the last few years. We also summarize the current knowledge regarding how it functions in gene expression. Finally, we will present new fields in which TREX seems to have an essential role.

## The composition of TREX

**Figure 2.**
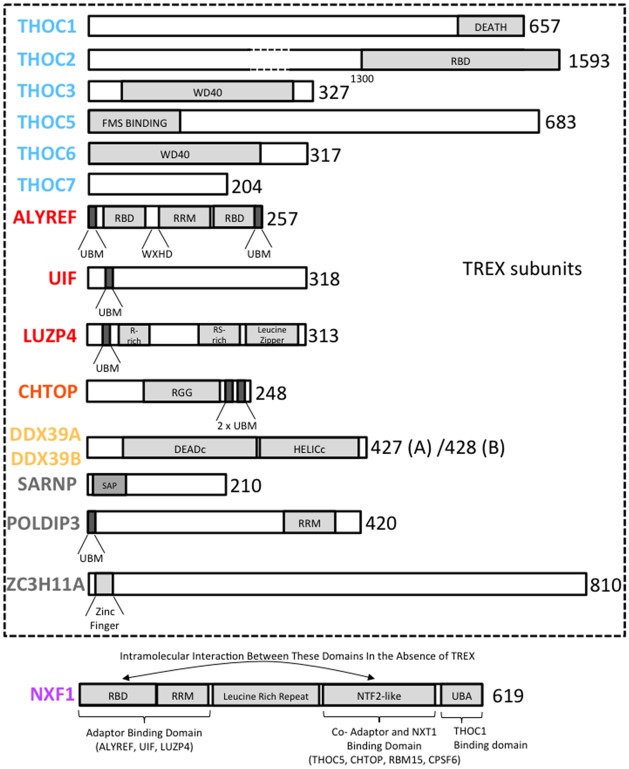
Schematics of the domain structures for mRNA export factors. The major characterized domains within TREX subunits and NXF1 are shown. Each protein and domain is drawn approximately to scale and the size of each protein in amino acids is shown on the right hand side. RBD, RNA-binding domain; R-rich, arginine-rich; RS-rich, arginine/serine-rich; RRM, RNA recognition motif (not necessarily involved in RNA binding). UBM, UAP56 (DDX39B)-binding motif; WxHD corresponds to the recently identified motif which binds EIF4AIII [12]. The other domains are all well characterized. Note that THOC2 is not drawn to scale due to its size.

TREX consists of a stoichiometric salt-resistant hexameric core called THO, consisting of THOC1, 2, 5–7 and Tex1 [10] together with a group of additional proteins. These can come together in different combinations to make alternative forms of TREX [14–19] (Figure 2 and Table 1). The THO subunits were originally linked to mRNP biogenesis and export through their mutant phenotypes in *S. cerevisiae* [31,47,48]. Subsequently, their existence as a stable THO complex was discovered by their ability to suppress the transcriptional defects of the Hpr1 mutant by overexpression [34]. The genetic and physical interaction of THO with the DEAD-box RNA helicase Sub2p and the RNA export adaptor Yra1p and equivalent biochemical interactions among the human orthologs led to the discovery of the conserved TREX complex [6]. Additional mammalian TREX subunits have been identified through molecular associations and functional characterization through depletion and overexpression effects [10,14,17,24]. TREX subunits have discrete functions with diverse cellular roles, coming together to form this influential complex in the cell and the *curriculum vitae* for each subunit is presented below.

**Table 1:** Composition of the TREX complex

	Human name	Alternative names	Cellular function	Yeast orthologs	kDa	Functional features	Key references			Link to BioGRID
THOsubunits	THOC1	hHpr1, p84, p84N5, N5	Apoptosis regulator	Hpr1p	76	Death domain, Rb-interacting factor	[20]	[21]	[196]	BIOGRID ENTRY
	THOC2	hRlr1	THO scaffold protein	Tho2p, Rlr1p	183	Coiled-coil domains, lysine-rich region	[31]	[15]	[22]	BIOGRID ENTRY
	THOC3	Tex1, hTrex45		Tex1p	39	WD40 domains	[15]	[10]		BIOGRID ENTRY
	THOC5	fSAP79, Fmip	Export co-adaptor		79	Leucine zipper, Fms-binding domain	[23]	[10]	[24]	BIOGRID ENTRY
	THOC6	fSAP35, Wdr58			38	WD40 domain	[10]	[15]		BIOGRID ENTRY
	THOC7	fSAP24, Nif3l1bp1			24	Coiled-coil domain, Leucine zipper	[10]			BIOGRID ENTRY
TREXsubunits	DDX39B	Uap56, Bat1, P47	Splicing factor, EJC-associated protein, RBP loading factor	Sub2p	49	ATP-dependent DEAD/DEAH-box RNA helicase	[25]	[6]	[26]	BIOGRID ENTRY
	DDX39A	Ddx39, Urh49	Ddx39b paralogue, Putative EJC-associated protein		49	ATP-dependent DEAD/DEAH-box RNA helicase	[27]	[28]		BIOGRID ENTRY
	ALYREF	Aly, Ref, Bef, Thoc4	Export adaptor, EJC-associated protein	Yra1p, She11p	27	RRM, Nxf1-binding, Ddx39-binding, UBM sequence	[29]	[6]	[30]	BIOGRID ENTRY
	UIF	Fyttd1	Export adaptor, EJC-associated protein		37	Functional homology with Alyref	[17]	[186]		BIOGRID ENTRY
	LUZP4	Hom-Tes-85, CT-8	Export adaptor, EJC-associated protein		36	Functional homology with Alyref, RS-dipeptides, Leucine zipper	[33]	[19]		BIOGRID ENTRY
	CHTOP	Srag, C1orf77, Fop	Export co-adaptor, EJC-associated protein		26	PRMT1-interaction domain, RGG box	[35]	[36]	[16]	BIOGRID ENTRY
	SARNP	Cip29, Tho1, Hcc-1		Tho1p	24	SAP domain	[37]	[14]		BIOGRID ENTRY
	POLDIP3	Skar, KIAA1649, PDIP46EJC-associated Protein			46	UBM-like sequence, RRM	[38]	[18]		BIOGRID ENTRY
	ZC3H11A	Zc11a, ZC3HDC11A			89	Zinc finger protein	[14]	[18]		BIOGRID ENTRY
	ERH	dDroer			12	Generally uncharacterized	[197]	[14]	[39]	BIOGRID ENTRY
TREX-associated proteins	NXF1	Tap	RNA export receptor	Mex67p	70	Arginine-rich RBD, pseudo-RRM, NTF2-like domain, UBA	[40]	[41]	[42]	BIOGRID ENTRY
	NXT1	p15	Required for Nxf1 stabilization and mRNA export	Mtr2p	15	Stabilizes and binds to NXF1's NTF2-like domain	[198]	[199]	[43]	BIOGRID ENTRY
	ZC3H18	Nhn1	NEXT complex component		106	CCCH-containing, Zinc-finger protein	[200]	[44]	[45]	BIOGRID ENTRY
	SRRT	Ars2	pri-miRNAs processing, CBC effector in RNA 3′ processing		100	Cap-binding protein	[201]	[202]	[203]	BIOGRID ENTRY
	NCBP3	C17orf85, Elg	Involved in RNA export upon viral infection		71	Cap-binding protein, forming an alternative CBC with Ncbp1	[204]	[14]	[46]	BIOGRID ENTRY
	NCBP1	Cbp80	Transcription elongation, RNA export, RNA stability	Cbc1p	71	Cap-binding protein, forming the CBC with Ncbp2	[205]	[11]	[15]	BIOGRID ENTRY

### THO

Human THOC1 is the ortholog of the yeast protein Hpr1 [6,15] and was originally discovered by its association with the tumor suppressor retinoblastoma protein (pRB) [20]. THOC1 is involved in cell cycle regulation and inducing p53-independent apoptosis through its death domain (Figure 2) [49–52]. THOC2 is the human ortholog of the yeast Tho2 protein and, as the largest subunit of TREX, is proposed to act as a scaffold for the formation of the complex [53]. THOC2 is an essential component for maintaining TREX function in humans [15,42], and mutations in the *THOC2* gene are associated with intellectual disabilities [22,54]. The conserved THOC3 (hTEX1) protein has been recently established as a component of the THO subcomplex in TREX and contains WD40 repeat motifs that allow multiple protein interactions (Figure 2) [10,15]. THOC5, 6 and 7 were originally identified as part of THO in human and *Drosophila* cells, but have no known yeast orthologs [7,10,24]. Mammalian THOC5 is a cytoplasmic substrate for the macrophage-colony stimulating factor receptor termed FMS [23] and is directly involved in the differentiation of macrophages [55], and a role in hematopoiesis and cancer has been established [56]. The THO complex is implicated in embryonic stem (ES) cell self-renewal, being responsible for the export of pluripotency transcripts in mouse ES cells [57]. THOC7 is devoid of a nuclear localization sequence but forms a tight complex with THOC5, allowing the heterodimer to be translocated to the nucleus [9]. THOC3 and THOC6 contain WD40 repeat motifs (Figure 2) commonly used as protein interaction domains, which may contribute to THO assembly.

### DDX39B

DEAD-box protein 39B (DDX39B), widely known as U2AF65-associated protein 56 (UAP56), is the metazoan ortholog of the yeast Sub2p protein. First discovered as an essential factor for pre-mRNA splicing through its association with U2AF2 [25], this RNA-stimulated ATPase and DEAD-box RNA helicase promotes spliceosome assembly [26,58]. DDX39B is present on pre-mRNAs during splicing [15,59] and is required for the subsequent recruitment of the TREX subunit ALYREF to both spliced and intronless mRNAs [59,60]. On binding ALYREF, the RNA bound to DDX39B is transferred to ALYREF [16]. This is a part of an orchestrated sequence of RNA handover events that occur in nuclear mRNA export, whereby the mRNA acts like a baton in a relay race. DDX39B accompanies the mRNP to the nuclear pore, but is eventually displaced before nucleocytoplasmic translocation [61]. The displacement is triggered by recruitment of the mRNA export receptor NXF1, which binds to ALYREF in a mutually exclusive manner with DDX39B (Figure 1) [30]. In mammalian cells, a paralog of DDX39B exists, DDX39A, which has some functional redundancy with DDX39B, because depletion of both helicases is required to efficiently block mRNA export [17,27]. However, different groups of mRNAs have their export affected following knockdown of DDX39A or B, indicating that the two helicases most likely assemble into subtly different TREX complexes acting preferentially on specific groups of mRNAs [28].

### ALYREF

ALYREF was originally described as a transcriptional coactivator of the T-cell receptor α gene by binding LEF-1 and acute myeloid leukemia (AML)-1 transcription factors to form an enhancer-stimulating complex [29]. It was subsequently characterized as a molecular chaperone capable of stimulating the dimerization of basic leucine-zipper transcription factors, thereby inducing their DNA-binding activity. These studies also showed that ALYREF has a propensity to form an oligomeric complex *in vitro* with a molecular weight of 460 kDa corresponding to 14 ALYREF monomers [62]. ALYREF contains N- and C-terminal transient helices, known as the UAP56-binding motif (UBM), which are necessary and sufficient for interaction with DDX39A/B [17] (Figure 2). It also has a central RNA recognition motif (RRM), which binds RNA weakly through loops 1 and 4 [63]. The RRM is flanked by two unstructured arginine-rich regions that are the principal RNA-binding sites. Such separate RNA-binding domains set within unstructured flexible peptides [63] would provide ALYREF with the potential to bridge interactions between separate parts of an mRNA, which is consistent with the RNA annealing activity observed for its yeast ortholog, Yra1 [64]. Such an activity suggests that ALYREF may play an important role in packaging of the mRNP, which adopts a compact crescent-shaped structure during translocation from the site of synthesis to the nuclear pore [65]. The arginine-rich regions are also the principal sites for interaction with the mRNA export receptor NXF1, and binding of NXF1 to ALYREF triggers transfer of RNA from ALYREF to NXF1 [30]. Arginines within the RNA-binding region of ALYREF are methylated. These modifications have been shown to reduce the ability of ALYREF to bind RNA which is important for the handover of RNA from ALYREF to NXF1 [66]. ALYREF binds phosphoinositides *in vitro* and mutations in the N- and C-terminal arginine-rich regions alter this binding*.* However, whether this association is physiologically important remains unclear since the mutations that alter phosphoinositide binding, leading to altered ALYREF localization *in vivo*, may also have altered its RNA-binding activity [67]. Like DDX39A/B, ALYREF is displaced from the mRNP before nucleocytoplasmic translocation [61,68]. ALYREF is involved in the export of both spliced and intronless mRNAs [69], and RNA interference (RNAi)-mediated knockdown of ALYREF leads to disruption of mRNA export for around 3900 mRNAs in 293T cells [70]. Functional redundancy between ALYREF and other mRNA export factors such as UIF (described below) may account for the relatively small number of mRNAs whose export is affected following ALYREF RNAi.

### UIF

UIF (also known as FYTTD1) was first identified using a BLAST search with the ALYREF UBM and, although it harbors this motif, it has relatively little additional homology with ALYREF and other mammalian proteins outside this peptide. UIF functions in a redundant manner with ALYREF and knockdown of both proteins leads to a strong mRNA export block in mammalian cells. Knockdown of ALYREF also leads to a large up-regulation of UIF levels in cells, which probably accounts for the modest block in mRNA export seen following ALYREF RNAi [17]. Although UIF associates with ALYREF, this interaction is RNA-dependent, whereas the association with other TREX subunits is not [17]. Thus, UIF may form an alternative TREX complex without ALYREF and the RNA-dependent association with ALYREF may arise because multiple TREX complexes are loaded onto an mRNA.

### LUZP4

LUZP4 was originally characterized as a cancer/testis antigen [33], which represents a group of poorly characterized proteins whose expression is normally restricted to testis but are frequently up-regulated in cancer cells. Subsequently, a BLAST search revealed that LUZP4 also carries a UBM (Figure 2) and acts as an mRNA export factor associating with TREX subunits and complementing ALYREF RNAi *in vivo*. While the expression of LUZP4 is normally restricted to testis, it is up-regulated in melanoma cancer cells where it is required for their survival [19].

### CHTOP

CHTOP was originally identified as an RNA-binding protein that regulates the cell cycle [71]. It was subsequently shown to be a binding partner for protein arginine methyltransferase 1. It is involved in the expression of the γ-globin gene and is recruited to the estrogen target gene, pS2, in breast cancer cells [35,72]. Its role in gene expression is further shown by its presence in the ‘five friends of methylated CHTOP’ desumoylation complex that is recruited to the Zbp-89 transcription factor during transcriptional activation [36]. The direct function of CHTOP in transcriptional regulation is akin to that of ALYREF. Therefore, CHTOP may also be key in linking the stages of transcription, pre-mRNA processing and export as a component of TREX. CHTOP contains two copies of the UBM found in ALYREF, UIF and LUZP4 (Figure 2) and is capable of stimulating the ATPase and helicase activities of DDX39A/B. Both ALYREF and CHTOP are dependent on DDX39A/B for efficient loading onto mRNA *in vivo* [16].

### SARNP

SARNP (also known as CIP29) was first identified as an EPO-stimulated cytokine-induced protein involved in cell cycle progression [37]. The small protein contains a SAP DNA-binding motif (Figure 2), suggesting a possible role in direct transcriptional regulation and it was subsequently identified as a TREX component [14]. Its yeast ortholog, Tho1, was identified alongside Tho2 as suppressing transcriptional defects of *hpr1* mutants [31], forming the initial connection to TREX [31,73]. SARNP forms an ATP-dependent trimeric complex with DDX39A/B and ALYREF [14]. Moreover, SARNP stimulates the ATPase and helicase activities of DDX39A/B [16,74]. The plant ortholog of SARNP is known as MOS11 where it has also been shown to facilitate mRNA export [75].

### POLDIP3

POLDIP3 was identified as an S6 kinase substrate with homology to ALYREF, which contributes to cell growth regulation [38]. It localizes to nuclear speckles, but also enhances translation [76], so is likely to play a role in many stages of mRNP biogenesis. It associates with DDX39A/B and TREX in an ATP-dependent manner and its over expression leads to retention of poly(A)^+^ RNAs in nuclear speckles [18].

### ZC3H11A

ZC3H11A co-purifies with multiple other TREX subunits [14] and recent work indicates that, similar to POLDIP3, it associates with TREX and DDX39A/B in an ATP-dependent manner. Knockdown of ZC3H11A by RNAi leads to a strong nuclear accumulation of mRNA [18]. Together, these data suggest that ZC3H11A is a bone fide TREX subunit, required for efficient mRNA export.

## Other proteins associated with TREX

Using immunoprecipitation with antibodies to THOC2, SARNP and DDX39A/B, a group of core proteins that associate with all three TREX subunits were identified using mass spectrometry. These included CBP80, CBP20, SRRT and NCBP3 (ELG), which all associate with the 5′ cap on mRNA and ERH [14]. ERH has multiple roles in the cell including roles in splicing and the cell cycle (reviewed in ref. [39]) and is a binding partner for POLDIP3 [77] and this may account for its association with TREX.

## Differences in TREX between species

The two major systems which have been used to study TREX are yeast and mammalian. While TREX is clearly conserved between these organisms (Table 1), there are also some important differences. For example, there are no known yeast orthologs for CHTOP, POLDIP3 and ZC3H11A. There is also divergence among the THO subunits. THOC1, 2 and 3 have orthologs in the two systems, but THOC5, 6 and 7 are specific to higher eukaryotes [7], with the yeast THO complex having two alternative subunits, Mtf2 and Thp2 [6]. Humans have a single ALYREF gene, whereas mice and yeast have two orthologous genes. UIF is conserved in mammals, but is absent from yeast and other higher eukaryotes such as *Drosophila* and *Caenorhabditis elegans*. LUZP4 is conserved in vertebrates but absent in other metazoans. These important species differences, with an expansion of TREX subunits during evolution, probably reflect the diverse biological roles that TREX plays particularly in multicellular organisms.

## Recruitment of TREX to mRNA through transcription and RNA processing

### Transcription

As a dynamic complex, TREX components are recruited to the developing mRNP at various stages of biogenesis. Chromatin remodeling and subsequent transcription form the first phase of mRNA generation. UIF is recruited to the transcription complex and the nascent RNA by association with the SSRP1 subunit of FACT [17], a histone chaperone and transcription elongation factor that remodels the H2A:H2B histone dimer, so RNA Pol II can transcribe the gene [78]. ALYREF is recruited by another histone chaperone Spt6, which works alongside FACT to remodel H3:H4 associated nucleosomes during transcription [79]. Spt6 binds the carboxy-terminal domain (CTD) of the large subunit of RNA Pol II and recruits IWS1. IWS1 in turn recruits ALYREF to actively transcribing genes [80].

The yeast TREX complex associates directly with the Pol II CTD; however, the interaction is partially dependent on the presence of nascent RNA [81,82]. This mechanism may ensure that TREX only associates with transcribing Pol II. Ser2 phosphorylation of the Pol II CTD is required for TREX binding and its occupancy on genes mirrors Ser2 phosphorylation, increasing in a 5′ to 3′ direction. When the increased recruitment of TREX towards the 3′-end of a gene is impaired, this specifically hinders the expression of long genes [81]. As TREX assembles on nascent RNA, there is a possibility that the CTD could become depleted of TREX subunits during transcription and this would be particularly acute on long genes. Therefore, Ser2-dependent enhanced recruitment of TREX to the CTD may ensure that it does not become depleted of TREX subunits during transcription of long genes. Interestingly, Mex67, the yeast ortholog of NXF1, is also necessary for Hpr1 (THOC1) stabilization during transcription [83], demonstrating the feedback and coordination between transcription and export steps.

The Prp19 complex, which functions in splicing, transcription and transcription-coupled DNA damage repair [84], is also required for efficient recruitment of TREX to transcribed genes in yeast. A mutation in the Syf1 subunit of the Prp19 complex results in less Hpr1, Sub2 and Yra1 associated with genes, especially at the 3′-end, suggesting that Prp19 acts to recruit TREX co-transcriptionally [85]. The human PRP19 complex associates with U2AF2 and together they promote Pol II CTD-dependent splicing activation [86]. Moreover, the PRP19 complex associates with TREX in human cells [14]. U2AF2, which directly binds DDX39A/B, may provide the link between TREX and the PRP19 complex, facilitating co-transcriptional recruitment of TREX to transcribed genes. An increasingly recurrent theme in gene expression is the coupling of each stage and reciprocal relationships between each step. It appears that this is also the case for transcription and export because TREX is required for efficient transcription elongation, particularly of long genes [21,87]. ALYREF affects transcription of a subset of genes [70], suggesting a reciprocal coupling between transcription and export via ALYREF, as it has been observed for other steps in the gene expression pathway [88].

### 5′ Cap formation

The NCBP1 (CBP80) and NCBP2 (CBP20) heterodimer forms the CBC, which associates with the 5′ cap. TREX is recruited to the 5′ cap by direct NCBP1–ALYREF and NCBP1–THO interactions [11,15]. Interestingly, SRRT also co-purifies with TREX [14]. SRRT associates with the CBC, the nuclear exosome targeting complex (NEXT) and together with ZC3H18 forms the CBC–NEXT complex [45]. ZC3H18 has independently been shown to recruit TREX to the hepatitis B virus post-transcriptional regulatory element and promote export of intronless mRNAs [44]. NEXT consists of three subunits, hMTR4, RBM7 and ZCCHC8, and multiple TREX subunits (ALYREF, CHTOP, THOC1,2,3,5,7, POLDIP3, UIF and DDX39A) co-purify with hMTR4 [45]. This raises the possibility that TREX bound to MTR4 might regulate the assembly of CBC–NEXT and the recruitment of the exosome. In this way, the assembly of TREX on the 5′ cap may be a critical decision point regarding whether an mRNA is degraded or exported during nuclear quality control. NCBP3 has recently been characterized as an alternative binding partner for NCBP1 at the cap, which can replace NCBP2. NCBP3 knockdown leads to a clear nuclear accumulation of mRNA, establishing its role in mRNA export. It also appears to act preferentially during times of stress such as during viral infection [46]. The earlier observation that NCBP3 associates with TREX [14] may account for the role of NCBP3 in mRNA export, by providing the means to ensure TREX recruitment to the 5′ end of mRNAs and promoting efficient mRNA export in times of stress. The length of an RNA determines its export pathway and RNAs shorter than 300 bases generally do not utilize the TREX:NXF1 pathway. This choice is determined early during transcription when hnRNP C associates with the 5′-end of the nascent RNA if it is long enough. hnRNP C binding to the 5′-end of the nascent RNA prevents recruitment of alternative export factors such as PHAX. This directs the RNA to the mRNA export pathway, whereby TREX probably displaces hnRNP C from the cap proximal region [89].

### Splicing

It has long been known that the presence on an intron enhances gene expression from an otherwise intronless cDNA and that splicing enhances mRNA export [90]. Using *in vitro* splicing reactions, it was shown that recruitment of human TREX is splicing-dependent [10,59]. The splicing dependence may be linked to the fact that DDX39A/B is involved in spliceosome assembly and additionally binds U2AF2, which recognizes the polypyrimidine tract within introns, early during spliceosome assembly. U2AF2 also co-operates with DDX39A/B to regulate nuclear retention of pre-mRNA [91]. Therefore, rearrangements of the U2AF2:DDX39A/B axis during splicing might couple TREX assembly on spliced mRNA with release of pre-mRNA retention signals. Interestingly, intron status is significant in yeast, despite direct co-transcriptional recruitment, as Yra1 is recruited preferentially to spliced mRNA [92]

The ability of TREX to be loaded onto spliced mRNA *in vitro* occurs in the absence of transcription and 3′-end cleavage/polyadenylation [10]. Furthermore, approximately 80% of splicing occurs co-transcriptionally in human cells [2], and so a considerable amount of TREX may be loaded onto mRNAs prior to 3′-end processing. Co-transcriptional splicing is thought to occur largely in regions of decompacted chromatin at the periphery of, or within, nuclear speckles. Moreover, TREX subunits interact *in vivo* on the periphery of nuclear speckles [93], suggesting that this is a major site for co-transcriptional TREX assembly. In contrast, post-transcriptional splicing appears to be largely restricted to nuclear speckles [2] and post-splicing release of mRNAs from speckles is dependent on TREX subunits [94].

Following splicing, the EJC is loaded onto the mRNA at a canonical position 24 bases upstream of the splice junction [95], furthermore the EJC co-purifies with TREX subunits [96,97]. These data led to the suggestion that the EJC provides a binding platform for mRNA export factors including the TREX subunit ALYREF and the mRNA export receptor NXF1 [96]. More recently, a specific sequence motif has been identified within ALYREF (WxHD) (Figure 2) that facilitates its interaction with the EJC subunit EIF4AIII. Thus, the EJC and CBC provide a stable binding platform for TREX on the 5′-end of mRNAs *in vitro* [12]. The involvement of the EJC in TREX loading is consistent with the recent observation that the EJC assembles on mRNAs in regions surrounding the nuclear speckles, termed perispeckles [98] (Figure 1), where TREX subunits also interact [93]. Whether mammalian TREX is loaded onto internal mRNA sites around EJCs *in vivo* remains to be seen. In yeast, this appears to be the case, with TREX subunits bound along the body of mRNAs [92,99].

Despite the central importance of splicing for normal mRNA export, when splicing is inhibited by drugs such as spliceostatin, this leads to the leakage of pre-mRNA to the cytoplasm [100]. However, the overall amount exported is very small and the vast majority of unspliced pre-mRNA is retained in the nucleus when observed by fluorescence *in situ* hybridization [101]. Nevertheless, since some pre-mRNA does leak to the cytoplasm, this may indicate that export factors can be loaded onto unspliced pre-mRNA *in vivo.* Thus, one of the key effects of splicing in mRNA export may be to ensure nuclear retention of pre-mRNA already loaded with export factors.

While intron removal is important for the export of spliced mRNAs, TREX subunits also associate with intronless mRNAs [69] and the association between CBC and TREX is important for their export [102]. Interestingly, U2AF2 also promotes intronless mRNA export in *Drosophila* [103], which may be connected with its ability to bind DDX39B. Specific internal RNA elements have been identified which promote intronless mRNA stability and export [104,105]. These elements bind U2AF2, PRP19 complex and TREX subunits and, therefore, may act as surrogates for splicing on intronless mRNAs. The recruitment of TREX to internal sites on mRNAs may be important to ensure the binding of multiple copies of the export receptor NXF1, ensuring smooth passage of the elongated mRNP through the nuclear pore. While TREX can function as a binding platform for NXF1, some intronless mRNAs appear to utilize additional strategies to ensure NXF1 recruitment. For example, histone mRNAs are bound by SR proteins that can in turn directly recruit NXF1 in a manner similar to ALYREF [106–108].

### 3′-end processing

A necessary pre-requisite for mRNA export from the nucleus to the cytoplasm is the release of mRNA from the DNA template, which is coupled to pre-mRNA 3′-end processing [3]. In yeast, two key proteins, Pcf11 and Clp1 come together to cause pre-mRNA cleavage. Pcf11 is recruited early during transcription through the Pol II CTD and also recruits Yra1 at the 3′-end of genes. Clp1 recruitment to Pcf11 triggers displacement of Yra1 from Pcf11 [13]. The ability of Yra1 to regulate accessibility of Clp1 to Pcf11 means that it can influence the site of cleavage/polyadenylation within a pre-mRNA, particularly at genes with divergent efficiency elements [109]. The displacement of Yra1 from Pcf11 by Clp1 is enhanced by the presence of Sub2, suggesting that the process is coordinated with loading Yra1 onto mRNA. Intriguingly, in both *tho* and *sub2* mutants, a stalled intermediate in mRNP biogenesis is created in which nuclear pore components and polyadenylation factors remain associated with chromatin in what is known as a ‘heavy chromatin’ fraction [110]. Together, these data indicate that TREX plays a key role in 3′-end processing and the concomitant release of the mRNP from chromatin.

The PCF11-ALYREF interaction is conserved in humans [13] and DDX39B has been found to be present in a highly purified 3′-end processing complex [111]. Thus, poly(A) site choice in mammalian cells is also likely to be governed by ALYREF, CLP1, DDX39A/B and PCF11, through similar mechanisms to those used in yeast. Additionally, THOC5 binds two 3′-end processing factors, CPSF100 [112] and CPSF6 [113]. Through these interactions, THOC5 can regulate poly(A) site choice for specific genes. In the case of CPSF6, THOC5 directs its early recruitment to transcribed genes and, following THOC5 or CPSF6 loss, proximal poly(A) sites are preferentially used [113]. A further twist in the ‘tail’ arises from recent studies on CDK11 that triggers Ser2 phosphorylation of the Pol II CTD [114]. Ser2 phosphorylation peaks at the 3′-end of genes and this stimulates the recruitment of 3′-end processing factors to the gene. TREX forms a stable complex with CDK11 and is required for its recruitment to the 3′-end of HIV 1 genes and subsequent CTD Ser2 phosphorylation. Therefore, TREX orchestrates 3′-end processing via CDK11 on HIV transcripts, but the extent to which TREX works with CDK11 on cellular mRNAs remains to be seen. In summary, TREX acts at many points in the evolution of the 3′-end of mRNA through interactions with multiple components of the transcription and 3′-end processing machinery. In turn, 3′-end processing, a necessary pre-requisite for mRNA export, no doubt allows the evolution of TREX to a state where it can subsequently trigger export of the fully processed mRNA.

## Integrating mRNA processing with TREX assembly

**Figure 3.**
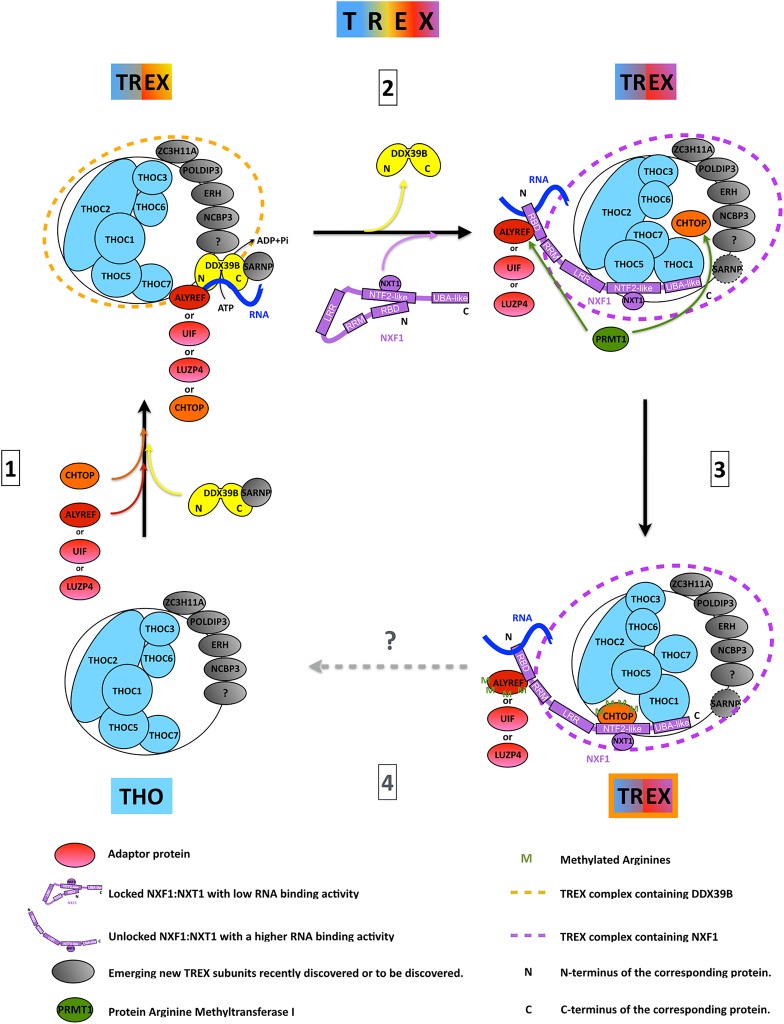
The life cycle of the TREX complex. The THO complex constitutes a salt-resistant core of tightly associated proteins (light blue). During gene expression, it dynamically associates with a variety of proteins (red, orange, yellow, purple and gray) to form the TREX complex, whose composition evolves during mRNP formation. THO is able to recruit the adaptor proteins (red), the co-adaptor CHTOP and the RNA helicase DDX39B. The DDX39B RNA helicase is thought to use rounds of ATP hydrolysis to load adaptors and co-adaptors onto the RNA. In turn, they recruit the mRNA export receptor NXF1:p15, which displaces DDX39B. While the co-adaptor THOC5, as a part of THO, is likely to associate early with the NTF2L domain of NXF1, arginine methylation of the second major co-adaptor CHTOP is a prerequisite for it to bind that same domain. This suggests that rearrangements occur within TREX. It is currently unclear whether the additional subunits (gray) are all part of the same TREX complex or belong to variants of a remodeled TREX complex. It is also unknown whether the same THO complex is recycled for further rounds of mRNP assembly (putative step 4).

**Figure 4.**
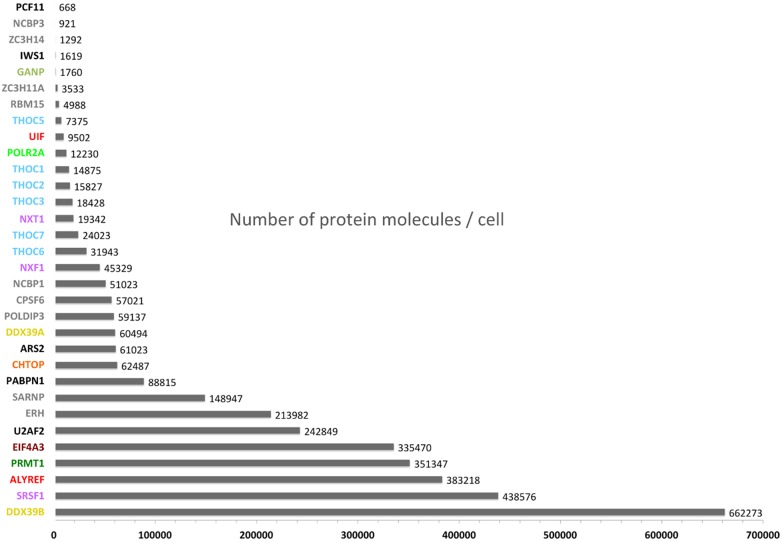
The number of protein molecules per cell for mRNA export and processing factors. This graph shows the average number of protein molecules per cell for a mouse NIH3T3 fibroblast cell line. The data were extracted from ref. [115].

The early assembly of TREX subunits probably occurs on the RNA Pol II CTD through multiple mechanisms, including direct binding of subunits to the Ser2 phosphorylated CTD and indirectly via the PRP19:U2AF2 complex. Subsequently, TREX subunits transfer to the nascent mRNP. This process is driven in part by the RNA helicases DDX39A and B, which load export factors such as UIF, ALYREF, LUZP4 and CHTOP onto the mRNP, using the helicase ATPase cycle [14,16] (Figure 3). The assembly on the mRNP is driven by the major pre-mRNA processing events (capping, splicing and polyadenylation) that provide their own protein signatures such as the CBC and EJC. These protein signatures probably serve to stabilize TREX at multiple positions along the mRNP (Figure 1). Interestingly, subunits of the THO complex are largely devoid of RNA-binding activity in yeast with the exception of Tho2 [53], and in mammals, THOC5 binds RNA weakly [42]. In contrast DDX39A/B, ALYREF and CHTOP bind RNA avidly [16] and these may well provide the main connection between TREX and the mRNA. Whether all ALYREF and CHTOP molecules associated with the mRNP are also bound by THO is not clear. Analysis of the relative amounts of mRNA export and RNA processing factors in mouse cells shows that DDX39B, SARNP, ALYREF and CHTOP are present in considerable excess over THO subunits and the RNA Pol II large subunit (Figure 4). For example, there is 90 times more DDX39B than THOC5 in the cell [115]. While ALYREF, CHTOP, SARNP and DDX39B are all present in excess of the cap-binding protein NCBP1, THO subunits are present in substoichiometric amounts, with the most abundant THO subunit, THOC7 being present at a ratio of approximately 1:2 with NCBP1. These variations in the levels of TREX subunits in the cell may reflect differential recruitment of specific TREX factors to subsets of mRNAs. In favor of this hypothesis, depletion of THOC5 alters the export of approximately 2.9% of mRNAs in mouse cells and depletion of multiple THO subunits in *Drosophila* only effects export of approximately 20% of the transcriptome [7]. However, the restricted sets of mRNAs affected could also be caused by functional redundancy between export factors such as that seen between ALYREF and UIF [17]. THO may alternatively play its major role in export by chaperoning the recruitment of ALYREF, DDX39B, SARNP and CHTOP onto mRNA through its association with RNA polymerase II. Consistent with this model, THO levels are much more comparable with RNA Pol II large subunit levels (Figure 4) and in yeast, chromatin immunoprecipitation experiments reveal that THO associates with the majority of transcribed genes [116]. The two possibilities are not mutually exclusive, but what is clear is that there are likely to be subtly different forms of TREX associating on different mRNAs and within a single mRNP, there are likely to be varying flavors of TREX present. The >3 fold excess of DDX39B, ALYREF and SARNP, which form a stable ATP-dependent trimer [14] over the nuclear cap-binding proteins (Figure 4), indicate that the cell has the opportunity to recruit multiple copies of this trimer to a single mRNP. Thus, these proteins may play an important role in mRNP packaging as well as export, consistent with the RNA annealing activity of Yra1 (ALYREF) [64]. Finally, it is striking that PCF11 is such a low abundance protein relative to THO or other mRNA export factors, with approximately 1000 times more ALYREF present in the cell. This vast excess of ALYREF probably helps ensure that PCF11 remains saturated with ALYREF until 3′-end processing, thus guarding against inappropriate poly(A) site choice. The substoichiometric amounts of PCF11 with respect to RNA polymerase II may indicate that it is used on selective transcripts in mammalian cells.

## Biological roles of TREX

### mRNA export

TREX plays a central role in mRNA export and this activity is governed by its ability to act as a binding platform for NXF1 [42]. Nuclear export factor 1 (NXF1, also called tip-associated protein, TAP) was originally identified in a yeast two-hybrid screen to identify cellular partners of tyrosine kinase-interacting protein (Tip), a viral protein from Herpesvirus saimiri [117]. A link between NXF1 and mRNA export was revealed by a synergistic combination of yeast genetic studies and biochemical experiments performed in metazoan systems. The yeast protein Mex67p was first shown to be essential for bulk poly(A)^+^ RNA nuclear export, consistent with its nuclear localization, its ability to bind mRNAs *in vivo* and to contact the nuclear pore complex [118]. Concurrently, NXF1 was shown to promote nuclear export of retroviral transcripts by binding to RNA secondary structures called constitutive transport elements (CTEs) [119]. Subsequently, it was shown that Mex67p's function in mRNA export is conserved in metazoans through its ortholog NXF1 [40].

NXF1 is a multidomain protein (Figure 2), with each domain connected by flexible linkers. Extensive structural characterization has revealed an unstructured N-terminal region preceding the following folded domains: an RRM fold, a leucine-rich repeat, an NTF2-like (NTF2L) domain and finally a C-terminal UBA domain [43,120,121]. The NTF2L domain forms a tight complex with NXT1, which is essential for the stability and activity of NXF1 [43]. The NTF2L and UBA domains provide separate binding sites for FG repeat sequences found in the nucleoporins lining the nuclear pore. These FG repeat binding sites help direct NXF1 to the nuclear pore [43].

An additional conserved protein complex known as TREX-2, which consists of GANP, PCID2, DSS1 and ENY2 in mammals, is involved in the docking of NXF1 at the nuclear pore during mRNA export (Figure 1, [122,123]). Certain subunits of TREX-2 are stably associated with the nuclear pore, but whether this association requires on-going transcription is currently unclear since conflicting data exist in the literature [122,124]. Work on the yeast complex has shown that it is involved in the translocation of genes to the nuclear pore and provides a direct connection between a gene's promoter, transcription and the nuclear pore through the mediator complex [125]. Furthermore, the ENY2 subunit of TREX-2 is also a component of the SAGA transcription activation complex [126], and thus TREX-2 may use multiple connections with the transcription machinery to direct a gene to the nuclear pore. Studies in the human system have shown that GANP is involved in the selective export of specific mRNAs [127,128], particularly those involved in gene expression steps, including RNA processing, splicing and ribosome biogenesis. GANP uses its FG repeat-like domain to interact with the FG repeat-binding regions of NXF1 and stimulates a local high concentration of NXF1 at the nuclear pore [122]. Therefore, TREX-2 may provide a fast track for the export of certain transcripts by positioning the gene close to the nuclear pore and promoting loading of NXF1 onto the mRNP and subsequent export. The use of TREX-2 by certain genes may be important for cells to respond rapidly to cellular stresses [128].

A systematic study of the RNA-binding activity of NXF1 domains using UV cross-linking revealed that the major RNA-binding activity resides within the N-terminal unstructured region amino acids 61–118, with weaker RNA-binding activity identified within amino acids 1–60 and the RRM (amino acids 119–198) [30]. These studies are corroborated by *in vivo* mRNP capture assays, which mapped the mRNP-binding site for NXF1 to amino acids 61–140 [129]. The presence of an RRM has caused some confusion in the literature over the years regarding the NXF1 mRNA-binding activity. In fact, the major RNA-binding domain (RBD) corresponds to amino acids 1–118, not the RRM (amino acids 118–198). Moreover, the LRR, NTF2L and UBA domains have no RNA-binding activity when assayed by UV cross-linking [30], though weak RNA-binding activity has been detected for the NTF2L domain using electrophoretic mobility shift assays [130]. The NXF1 RBD is rich in arginines and mutation of 10 of these arginines prevents NXF1 mRNA binding both *in vitro* and *in vivo*. Moreover, a mutant form of NXF1, which carries the 10 arginines mutated to alanine, fails to function in mRNA export *in vivo* [30], establishing the RBD as the major determinant for mRNA binding. The binding of NXF1 to retroviral CTE elements appears to be fundamentally different from binding to mRNA and involves extensive interactions between the RRM, LRR and NTF2L domains of NXF1 [41,121,130]. Interestingly, the *NXF1* gene harbors a CTE-like structure within intron 10 and NXF1 binding to this sequence promotes the export of a retained intron transcript that encodes a short form of NXF1, though the function of this short form of NXF1 is unclear [131].

ALYREF binds to the N-terminal region of NXF1, including the RBD and RRM domains [132] (Figure 2). As ALYREF binds mRNA avidly, it was given the name mRNA export adaptor, because it was thought to bridge the interaction between RNA and NXF1. However, subsequent studies showed that when NXF1 binds to an ALYREF:RNA complex, the RNA is handed over to NXF1 [30] and ALYREF no longer binds RNA. This is consistent with biochemical studies, showing that the NXF1-binding site and RNA-binding sites on ALYREF overlap [63]. Additional mRNA export adaptors have been identified including various SR proteins, which, like ALYREF, use an arginine-rich peptide to bind the RBD + RRM domains of NXF1 [106–108]. A common feature of the RNA handover process from an export adaptor to NXF1 is that once the adaptor protein is bound to NXF1 it enhances NXF1 RNA binding activity [30]. The reasons for this were unclear until it was realized that NXF1 forms an intramolecular interaction between the RBD and the NTF2L domains which suppresses RNA binding (Figure 3) [42]. Thus, the NTF2L domain of NXF1 is autoinhibitory for mRNA binding driven by the RBD. ALYREF is involved in disrupting the NXF1 intramolecular interaction, which exposes the RBD for interaction with RNA. A second site for interaction between TREX and NXF1 involves the NXF1 UBA domain that binds THOC1 [42]. The third site is the NTF2L domain, which THOC5 binds on the opposite side to that bound by NXT1. The binding of ALYREF to NXF1 stimulates the binding of THOC5 to the complex; on the basis of this activity, THOC5 was named a co-adaptor for mRNA export [24]. The NXF1 RBD binds to the NTF2L domain at a site overlapping that bound by THOC5 and it is the combined action of ALYREF and THOC5 binding to NXF1 that exposes its RBD, allowing optimal RNA-binding activity [42]. Since NXF1 requires both an adaptor and a co-adaptor for optimal RNA binding, this raises the question of whether SR proteins function independently to recruit NXF1 to mRNAs, given they would only provide the adaptor function. Evidence from yeast suggests that SR proteins may function in collaboration with TREX during mRNA export [133–135], and this may be to ensure that a co-adaptor is available at such sites for optimal NXF1 RNA binding.

THOC5 is not the only co-adaptor protein that binds the NTF2L domain of NXF1; CHTOP, RBM15 and CPSF6 also bind this domain [16,136,137]. Similar to THOC5, CHTOP binding to NXF1 is stimulated by the presence of ALYREF [16]. CHTOP and THOC5 are found in the same TREX complex bound to NXF1; therefore, there is potentially sequential binding of these factors to NXF1 during mRNP maturation (Figure 3). The methylation of arginines within CHTOP is essential for its interaction with NXF1 [16] and methylation of arginines within ALYREF is required for RNA handover to NXF1 [66]. Therefore, arginine methylation plays a vital role in the activity of TREX during mRNA export.

An interesting feature of two of the known NXF1 co-adaptors, THOC5 and CPSF6, is their involvement in 3′-end processing and polyadenylation. Furthermore, NXF1 loss in cells leads to hyperadenylation of mRNA and loss of Mex67 in yeast leads to retention of the 3′-end processing factors Rna14 and Rna15 on the mRNA [138]. A further factor involved in the recruitment of NXF1 to the mRNP is ZC3H3 and loss of this factor leads to mRNA hyperadenylation, similar to that seen following NXF1 depletion [139]. These observations suggest that an adaptor, such as ALYREF, delivered via PCF11 and a co-adaptor, such as THOC5, delivered through interactions with 3′-end processing factors provide a key signal at the 3′-end of mRNAs. This signal is interpreted by NXF1 that subsequently binds to TREX, allowing correct evolution of the 3′-end processing complex to a state suitable for mRNA export.

Once NXF1 has been delivered to the mRNP by TREX, the mRNP is escorted to the nuclear pore [128]. Studies on *Chironomus tentans* have shown that ALYREF and DDX39B dissociate from the mRNP on the nuclear side or during translocation through the nuclear pore [61]. This is predicted to revert NXF1 to a low-affinity RNA-binding state and this may be an important prerequisite for its subsequent removal from the mRNP. Dbp5, alongside its ATPase activator Gle1 and inositol hexakisphosphate phosphate 6, work together with NUP159 to remodel the mRNP on the cytoplasmic side of the nuclear pore, triggering removal of NXF1 [140–142]. NXF1 is then reimported into the nucleus for subsequent rounds of mRNA export.

### piRNA biogenesis

While the majority of work on TREX has thus far focused on its role in mRNA export, recent studies indicate that it has additional roles in piRNA biogenesis. piRNAs are short RNA molecules produced in germ line cells with a major function in suppressing transposons during development. The first indication that TREX may be involved in piRNA biogenesis came from the observation that DDX39A/B co-localized at piRNA clusters with Rhino, which is required for transposon silencing [143]. Moreover, DDX39A/B mutations disrupted a cytoplasmic structure known as the nuage, which is rich in components of the piRNA processing machinery. Most recently, it has been shown that additional TREX subunits are involved in piRNA transcription and biogenesis, with TREX loading onto piRNAs being driven by a chromatin-associated protein, Cutoff [144]. However, it is not clear yet whether TREX plays a role in the transport of piRNA precursors from the nucleus to the cytoplasm.

### Genome stability, R-loops and cancer

During transcription, the nascent RNA can hybridize with the single-stranded template DNA present within the transcription bubble. This structure is known as an R-loop and it leads to the opposing DNA strand being left single-stranded, making it susceptible to cleavage which can eventually lead to various forms of DNA damage in cells [145,146]. TREX is intimately associated with both the RNA Pol II CTD and nascent RNA, leaving it poised to play a major role in sequestering the nascent RNA and preventing it from hybridizing to the template DNA strand. A large increase in R-loops is found in TREX-depleted yeast and human cells leading to far more DNA damage, transcription-associated recombination and DNA replication obstacles [87,116,147,148]. R-loops also form part of a natural process of genomic recombination to generate variation, such as for antibody class-switching in B-cells. THO depletion in murine cells results in an increased rate of class-switching as more R-loops are formed and consequently, a greater rate of recombination can occur [87]. An RNA/DNA helicase, SETX (senataxin; Sen1 in yeast), is required to resolve R-loop formation. Interestingly in yeast, Sen1 co-purifies with Yra1 (ALYREF) [149], raising the possibility that as well as packaging RNA to help prevent R-loop formation, ALYREF might also be involved in the regulation of SETX activity. A further role for TREX in maintaining genome stability arises from studies on IPMK, which is involved in inositol phosphate production in human cells. Loss of this kinase prevents the export of mRNAs encoding proteins involved in the DNA damage response. ALYREF is implicated in this process and thus, TREX maintains appropriate levels of proteins required to maintain genome stability by ensuring efficient export of their mRNAs [150]. Since TREX can associate with various different adaptor proteins such as UIF and LUZP4 (Figure 3), this raises the possibility that alternative forms of TREX promote the export of specific classes of mRNA [128].

Given the importance of TREX in maintaining genome stability, it is unsurprising that TREX has been implicated in many forms of cancer. CHTOP is recruited to the pS2 promoter, inducing the expression of estrogen target genes in breast cancer cells [35,36] and thereby maintaining growth of the tumor. CHTOP is also required for the expression of key genes required for the maintenance of tumor cells in glioblastoma [151]. THOC1 associates with the tumor suppressor pRB, resulting in the prevention of cell cycle arrest and THOC1-induced apoptosis [20,49,50]. This links THOC1 with tumor survival as mRNP biogenesis cannot occur efficiently enough to maintain rapid cell division in its absence [152,153]. The up-regulation of THOC1 and THO complex expression, readily seen in a range of cancer types, may correlate with tumor size and have a greater effect in hormone-dysregulated cancers [152–155]. Phosphorylation of THOC5 on tyrosine 225 promotes its incorporation into mRNPs and THOC5 phosphorylation by leukemogenic protein tyrosine kinases is increased in patients with chronic myeloid leukemia [156]. ALYREF also shows an altered expression pattern in various cancerous tissues, but is more frequently up-regulated [154,157]. Depletion of ALYREF results in the reduced metastatic capacity of human oral squamous cell carcinoma cell lines, demonstrating the increased TREX dependence in cancer cells. Interestingly, ALYREF depletion in this cell line results in increased levels of ‘metastasis modulating molecules’ RRP1B and CD82 that suppress the metastatic capacity of tumor cells [158–160]. ALYREF directly interacts with RRP1B, so the increased expression of ALYREF in cancer cells may titrate away RRP1B allowing metastasis to develop uninterrupted [157]. DDX39B depletion affects the expression levels of the tumor suppressor BRCA1 protein, which is important for the DNA damage response [28]. SARNP is up-regulated in cancer tissues [37,161], but a further link to AML has been made attributable to a translocation event creating an SARNP–mixed lineage leukemia (MLL) fusion protein [162]. As MLL does not have any intrinsic DNA-binding capability, the SARNP–MLL fusion may confer this action through SARNP's SAP DNA-binding motif (Figure 2), potentially resulting in altered gene expression, a hallmark of cancer development [163].

TREX seems to be directly involved in regulating the cell cycle, consistent with a role in proliferation and differentiation, and this activity probably correlates with the aberrant expression of TREX subunits in cancer cells. During mitosis, the cell moves through characteristic stages and TREX may function at the intermediate checkpoints. THOC1 and THOC2 influence the rate of proliferation, with THOC1 playing a more specific role in the G2-M checkpoint [50,54]. ALYREF is significant in the S and G2 phases where its expression seems co-ordinated with that of Cyclin A [67]. ALYREF also represses the activity of the E2F2 transcription factor when bound to the CHK1 promoter, preventing the expression of the CHK1 checkpoint kinase that induces cell cycle arrest and checkpoint activation [164]. Furthermore, DDX39B and THOC2 may play a role in chromosome alignment [165] and spindle assembly [166], though whether these are direct effects or indirect through failure to export crucial mRNAs remains unclear.

The numerous associations of TREX in cancer development could lead to new specific therapeutics. The accumulating evidence that cancerous tissues are more dependent on TREX to aid their survival and progression suggests that drugs to deplete TREX levels and/or activity could be a potential target for cancer therapy. The marked reduction in metastatic capacity and cancer cell proliferation following ALYREF [157], THOC1 [21,153] and LUZP4 [19] depletion further suggests that there may be therapeutic benefit in targeting TREX.

### Cellular differentiation

In addition to elevated TREX dependence in cancerous tissue, ES cells show a greater susceptibility to TREX loss. ES cells are pluripotent and derived from the inner cell mass (ICM) of the pre-implantation embryo at the blastocyst stage. Depletion of THO components at this stage results in embryonic lethality as the blastocyst cannot be effectively formed due to increased apoptosis of the ICM [56,57,167]. However, depletion of these components in differentiated adult cells does not result in immediate cell death [168] and THOC1 expression is decreased in nonproliferating cells [52], suggesting a greater dependency on TREX in undifferentiated cells early on in embryogenesis. Furthermore, the balance between differentiation and self-renewal within stem cells may be regulated by TREX through its function in mRNA export. Reduced levels of THOC2 and THOC5 during differentiation result in a decreased expression of self-renewal factors NANOG and SOX2, but an increased expression of differentiation markers GATA3 and BRACHURY [57,169]. THOC2 and THOC5 associate with differentiation marker mRNAs more readily than self-renewal mRNAs and the nuclear accumulation of NANOG and SOX2 mRNA suggests that this regulation occurs through mRNA export [57].

The differentiation and development of specific tissues show a greater demand for TREX. The levels of THOC5 and SARNP are greater in the human fetal liver, where hematopoietic cells differentiate [23], and SARNP is up-regulated in the adult bone marrow [37]. THOC5 is known to regulate differentiation of hematopoietic cells to various lineages [55] and may do so by regulating the expression of specific transcription factors involved in monocyte development [170]. The regulation of immediate early genes involved in macrophage differentiation is dependent on THOC5, perhaps linked to 3′-end processing of the transcripts [112]. Bone marrow and spleen cells are specifically affected following the loss of THOC5, resulting in death after a few days in mice [56,171], consistent with the loss of differentiating cells when THOC5 is depleted [172]. Conversely, THOC5 is dispensable in the terminally differentiated liver [172]. The small intestine is remarkably sensitive to the loss of TREX, presumably due to the presence of stem-cell niches in the intestinal crypt. THOC1 expression is higher in intestinal crypts harboring stem-cell niches [168], and THOC1 depletion results in degeneration of the small-intestinal epithelium and increased apoptosis of cells in the niche [168,172]. Interestingly, the stem-cell niches in the large intestine are not as affected by THOC1 depletion [168], demonstrating the tissue-specific effects of TREX loss as well as the increased requirement for the complex in differentiating tissues. Male gametogenesis requires rapid proliferation and stem-cell differentiation in the testes to produce sperm. Concurrent with increased TREX dependency in differentiating cells, fertility in male mice was greatly reduced when two hypomorphic THOC1 alleles were present [173]. Female mice also had reduced fertility when homozygous for the hypomorphic allele, most probably due to defects in the ICM during embryogenesis as mentioned above.

From studies in cancer, embryogenesis and tissue differentiation, the dependence on TREX for cellular survival seems to be increased in more rapidly proliferating and differentiating cells. Proliferating cells will have a higher rate of replication, transcription and therefore processing, export and translation to maintain cell division and induce differentiation. This increased burden on the gene expression machinery probably dictates the dependency on TREX that is central to this pathway.

### Neurodevelopment and neurodegenerative diseases

The role of TREX in neuronal disorders can be divided into two groups: direct mutations in TREX subunits, which trigger neuronal disease, and mutations in other genes, which lead to a potential dependence on TREX during the disease state. In the first class, missense mutations in the *THOC2* gene cause syndromic intellectual disability [22]. Additionally, a *de novo* translocation event in the vicinity of *THOC2* on the X-chromosome has been described in a child with cerebellar hypoplasia, ataxia and retardation [54]. The translocation of the PTK2 kinase gene generates a fusion transcript with THOC2 and leads to a decrease in expression of both encoded proteins. As inactivation of PTK2 alone does not cause this phenotype, the effects on *THOC2* are likely to be hugely influential in causing this disease, corroborated by *C. elegans* knockout studies showing impaired movement [54]. *THOC6* mutations have also been associated with intellectual disability, consistent with its high expression levels in the developing zebrafish brain [174]. The THOC6 mutation results in a mislocalization of the protein to the cytoplasm, suggesting that its nuclear role as a part of TREX is essential for proper neural and organ development. Furthermore, a homozygous mutation in a conserved region of *THOC6* is the likely cause of a disease with clinical features such as intellectual disability, brain malformation and renal and heart defects [175].

A second class of neuronal disease that indirectly depends on TREX is amyotrophic lateral sclerosis (ALS), associated with death of motor neurons. The most common familial form of ALS involves a GGGGCC repeat expansion within an intron of the *C9ORF72* gene. Following transcription, the repeat expanded pre-mRNA is exported to the cytoplasm where it forms RNA foci. Thus, the transcript overrides the normal nuclear retention mechanisms that keep pre-mRNA in the nucleus. The repeat expansion can also be translated in multiple reading frames in an ATG start codon-independent manner, leading to the production of toxic dipeptides (reviewed in ref. [176,177]). A survey of proteins that bind the repeat expansion identified multiple proteins including ALYREF, leading to the suggestion that excess ALYREF recruitment to the repeat expanded pre-mRNA, may stimulate its export [178]. A *Drosophila* model for neurodegeneration triggered by the C9ORF72 was recently used to carry out a genetic screen for suppressors and enhancers of neurodegeneration [179]. This screen identified a mutation disrupting ALYREF activity as a potent suppressor of neurodegeneration in this system and intriguingly identified CHTOP and NXF1 as enhancers. Furthermore, it was shown that the toxic peptides that are produced by the repeat expansion block mRNA export in human cells [179]. Therefore, in C9ORF72 disease, one unexpected reason for cell death may be the inhibition of expression of multiple proteins through a general mRNA export block. This export block is triggered by the inappropriate export of the pre-mRNA containing the repeat and this may involve the excessive recruitment of ALYREF to the repeat expansion.

### Viral replication

Viruses replicate by utilizing host cell machineries, which are essential for them to infect more cells and organisms. One such machinery is the NXF1–NXT1 mRNA export pathway that is employed to export viral mRNA, under the guise of a host mRNA. This ‘hijacking’ is conserved among viral species and subfamilies as it is used in Influenza [32,180,181], various Herpes strains and the Hepatitis B virus. The mRNA export pathway is typically hijacked by the expression of viral-specific export adaptors that serve to recruit TREX and NXF1 to viral mRNA. The Kaposi's Sarcoma-associated Herpes Virus and Herpes virus saimiri ORF57 are the most well studied of these adaptors, recruiting ALYREF to the viral mRNA to which ORF57 is bound [182–185]. The rest of TREX is also recruited, so the established RNA handover to NXF1–NXT1 can occur [184,186]. The Herpes simplex virus ICP27 [185,187], Epstein-Barr virus EB2 [188] and Human cytomegalovirus UL69 [189] proteins are functionally homologous to ORF57 as they similarly recruit ALYREF, the rest of TREX and NXF1 for viral RNA export. In some cases, DDX39B seems to have more of an influence on viral RNA export than ALYREF [189,190], supporting the role of the whole TREX complex in viral mRNA export and efficient viral replication. In addition, ALYREF may act to stabilize viral mRNAs at the 3′-end [191]. As well as viral export adaptors, elements in viral RNA act to recruit the host cell mRNA export machinery. The posttranscriptional regulatory element is necessary for efficient expression of Hepatitis B proteins and triggers recruitment of TREX [44]. Constitutive transport elements have been identified in a number of simple retroviruses, including Mason Pfizer monkey and Simian retrovirus, which directly bind NXF1 with high affinity, promoting export of viral transcripts [119]. Therefore, viruses have evolved a number of different strategies to hijack the cellular mRNA export machinery to ensure efficient expression of their own proteins.

### Plant development

Unsurprisingly, TREX seems to play a role in other RNA pathways. The existence and general function of TREX seems conserved in plants [8,75,192,193], and it also appears to play a role in the transport of small-interfering RNA precursors for long-distance gene-silencing effects [8,194]. siRNA interaction with AGO may require TREX to localize the siRNA, or to target the AGO protein necessary for gene silencing [8,194]. THO mutants disrupt these pathways differentially depending on the plant's developmental stage, consistent with human systems showing a greater dependence on TREX during embryogenesis and in differentiating tissues. TREX is implicated in other plant processes such as disease resistance and ethylene signaling, which is essential for development. Ethylene-induced senescence is increased in Hpr1 mutants, due to defective transcription of ethylene signaling and ribosomal protein genes [193,195]. The involvement of TREX in pathways other than mRNA export suggests that it may be a more general RNA trafficking module in the cell beyond plants, capable of influencing the localization of other RNA classes yet to be discovered.

## Discussion

**Figure 5.**
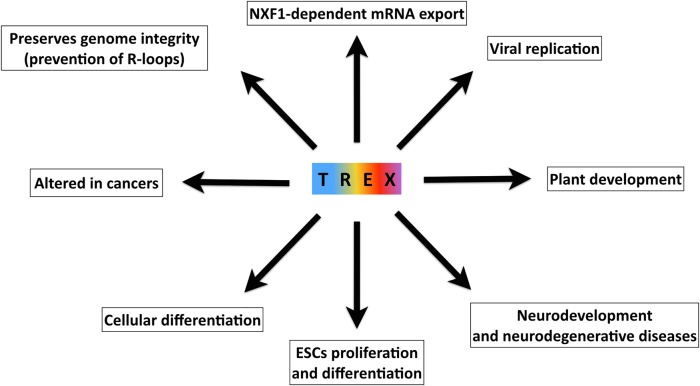
An overview of the biological processes involving TREX. ESC: embryonic stem cell.

The conserved TREX complex is highly integrated into cellular function and homeostasis (Figure 5), functioning throughout mRNA biogenesis and in many other cellular mechanisms. Its presence is necessary for effective transcription, processing and export as well as preventing DNA damage and maintaining differentiation during embryogenesis and in specific adult tissues. The dynamic nature of the proteins that associate with TREX is likely to provide the flexibility for the complex to carry out multiple functions and link mRNA generating and processing steps together. The less well-characterized members of TREX such as ERH, ZC3H11A and POLDIP3 are likely to reveal further functions of TREX and refine the functions already known. A key and important step in understanding mammalian TREX function in the future will be to elucidate its RNA substrates, transcriptome-wide, as has recently been done for yeast TREX [92,99]. The additional functions of TREX in the cell cycle, proliferating cells and the prevention of DNA damage must be investigated further, especially as understanding TREX could facilitate the development of novel drugs to treat cancer, ALS and viral infections.

## Abbreviations

ALS, amyotrophic lateral sclerosis; AML, acute myeloid leukemia; CBC, cap-binding complex; CTD, carboxy terminal domain; CTEs, constitutive transport elements; DDX39B, DEAD-box protein 39B; EJC, exon junction complex; ES, embryonic stem; EPO, erythropoietin; ICM, inner cell mass; MLL, mixed lineage leukemia; mRNP, messenger ribonucleoprotein; NEXT, nuclear exosome targeting complex; NMD, nonsense mediated decay; NTF2L, NTF2-like; NXF1, nuclear export factor 1; Pol II, polymerase II; poly(A), polyadenosine A; pRB, retinoblastoma protein; PRMT1, protein arginine methyltransferase 1; RBD, RNA-binding domain; RNAi, RNA interference; RRM, RNA recognition motif; TAP, tip-associated protein; TAR, transcription-associated recombination; Tip, tyrosine kinase interacting protein; TREX, TRanscription and EXport; UAP56, U2AF65-associated protein 56; UBM, UAP56-binding motif.
